# Inhibitory Potential of *Turbinaria ornata* against Key Metabolic Enzymes Linked to Diabetes

**DOI:** 10.1155/2014/783895

**Published:** 2014-06-24

**Authors:** P. S. Unnikrishnan, K. Suthindhiran, M. A. Jayasri

**Affiliations:** Marine Biotechnology and Biomedicine Laboratory, School of Biosciences and Technology, VIT University, Vellore 632007, India

## Abstract

One of the therapeutic approaches in treating diabetes is to reduce postprandial hyperglycemia by inhibiting major carbohydrate hydrolyzing enzymes. In the present study, crude extracts of marine seaweed, *Turbinaria ornata*, were tested for their antidiabetic potential using enzyme inhibitory assays (*α*-amylase, *α*-glucosidase, and dipeptidyl peptidase-IV). Among the tested extracts, methanol and acetone extracts showed significant inhibitory effects on *α*-amylase (IC_50_ 250.9 *μ*g/mL), *α*-glucosidase (535.6 *μ*g/mL), and dipeptidyl peptidase-4 (55.2 *μ*g/mL), respectively. Free radical scavenging activity of these extracts was analyzed using DPPH assay (65%). Extracts were tested for *in vitro* toxicity using DNA fragmentation assay, haemolytic assay, and MTT assay. None of the extracts showed toxicity in tested models. Furthermore, GC-MS analysis of lead extracts showed the presence of major compounds, hentriacontane, z, z-6, 28-heptatriactontadien-2-one, 8-heptadecene, and 1-heptacosanol. Our findings suggest that *Turbinaria ornata* can be used as a potential source for further *in vivo* studies in controlling hyperglycemia.

## 1. Introduction

Diabetes mellitus is a metabolic disorder which affects the endocrine system of the body, characterized by defects in carbohydrate, lipid, and protein metabolism [1]. Globally the frequency of disorder is rising gradually; patients suffering from this disorder are unable to turn out or respond properly to insulin produced in the body [2]. Control of postprandial hyperglycemia prevents chronic vascular complications, and it is a widely accepted goal for the management of type-2 diabetes [3]. *α*-Amylase and *α*-glucosidase are the two major enzymes which play a key role in the digestion of carbohydrates by hydrolysis of inner *α*-1, 4-glucosidic linkages in starch and several other polysaccharides and the uptake of glucose by intestinal *α*-glucosidases [4]. One of the effective strategies for the management of diabetes is by retarding the starch digestion rate by inhibiting these enzymes leading to the reduction of postprandial hyperglycemia in diabetic subjects [5]. A recent approach for the development of an antidiabetic drug is based on the development of incretin analogues and dipeptidyl peptidase-IV (DPP-IV) inhibitors which play a crucial role in the glucose homeostasis by promoting *α* and *β* cell function [6]. It also downregulates the gastric emptying and gastric acid secretion to reduce the postprandial glucose level [7, 8]. Currently, there are several commercially available DPP-IV inhibitors (sitagliptin, saxagliptin, and vildagliptin) to block DPP-IV action thereby prolonging the half-life and biological activity of incretin hormones [9]. On the other hand, some pharmacokinetics studies reveal that DPP-IV inhibitors, mainly vildagliptin, are not safe for the patients with severe liver problems [10, 11]. Similarly, other synthetic hypoglycemic agents (acarbose and voglibose) that inhibit *α*-amylase and *α*-glucosidase can cause hepatic and gastrointestinal disorders [12]. Experimental and clinical evidences show that hyperglycemia increases the production of free radicals and induces oxidative stress leading to various diseases like diabetes mellitus, cardiac disorders, inflammatory, and neurodegenerative diseases [13–15]. Hence, there is a need for new, safe, and efficacious antioxidant and antidiabetic agents.

Natural sources such as marine flora and fauna, bacteria, fungi, and higher plants are of new interest in the development of antidiabetic agents. Among them, marine seaweeds represent one of the richest sources of bioactive compounds which are widely used in pharmaceutical research [16–18]. Polyphenols and sulfated polysaccharides present in seaweeds have been proven for antiviral, antitumoral, anti-inflammatory, and anticoagulant activity [17–19]. Seaweeds contain prolific source of bioactive compounds which can be exploited for the treatment of major chronic diseases like diabetes through the inhibition of starch digesting enzymes and the regulation of glucose induced oxidative stress [20, 21].

In the present study, antidiabetic and antioxidant effect of five different solvent extracts of marine seaweed* Turbinaria ornata* has been evaluated.* T. ornata*, a wide spread species belonging to Phaeophyceae, is rich in fucoids and sulphated polysaccharides [22, 23]. It has been previously reported for its antioxidant, anti-inflammatory, and cytotoxicity activity on murine melanoma and colon cancer cells [23–25]. To the best of our knowledge, this is the first report on* T. ornata* elucidating three different antidiabetic mechanisms (*α*-amylase, *α*-glucosidase, and DPP-IV)* in vitro* and the extracts were also tested for their* in vitro* toxicity.

## 2. Materials and Methods

### 2.1. Chemicals

Porcine pancreatic *α*-amylase, dinitrosalicylic acid (DNSA), acarbose, p-nitrophenyl *α*-D-glucopyranoside, diprotin A (Ile-Pro-Ile), DPP-IV from porcine kidney, and Gly-pro-p-nitroanilide (GPPN) were purchased from Sigma, Bangalore, India. DPPH, butylated hydroxy toluene (BHT) was obtained from Himedia, Mumbai. *α*-Glucosidase and dimethyl sulphoxide (DMSO) were obtained from SRL, Mumbai.

### 2.2. Collection and Processing of Seaweed

Fresh brown seaweeds were collected from Mandapam coastal region (Latitude 9°17′′N; Longitude 79°11′′E), Gulf of Mannar, Tamilnadu, South India on low tide during October 2012. The voucher specimen was deposited in the Marine Biotechnology and Biomedicine Laboratory, VIT University. The seaweeds were cleaned and holdfasts were removed and stored at −20°C immediately. Before extraction, they were shade-dried, powdered, and stored in air-tight containers at −20°C.

### 2.3. Preparation of Seaweed Extracts

The dried seaweed samples (25 g) were milled and extracted using 250 mL of various solvents such as petroleum ether, benzene, ethyl acetate, acetone, and methanol for 24 h by using Soxhlet apparatus. Each filtrate was concentrated to dryness under reduced pressure using rotary evaporator (Super Fit, Rotavap, model, PBU-6, India). The samples were lyophilized by using freeze dryer (Lark, Penguin Classic Plus, India) and stored in a refrigerator at 2–8°C for use in subsequent experiments. Prior to experiments, various concentrations were prepared from 250 to 1000 *μ*g/mL.

### 2.4. Preliminary Phytochemical Screening

Preliminary phytochemical screenings were carried out as per the standard protocols of Harborne [26].

### 2.5. 2,2-Diphenyl-1-picrylhydrazyl (DPPH) Radical Scavenging Activity

Free radical scavenging activity was determined according to the method of Mensor et al. [27]. Briefly, 500 *μ*L of 0.3 mM alcoholic solution of DPPH (Himedia, India) was added to 2.5 mL of test samples at varying concentrations (250–1000 *μ*g/mL). After incubation of samples in the dark for 30 minutes, the optical density was measured at 518 nm in the UV-visible spectrophotometer (Systronics AU-2700, India) using methanol as blank and synthetic antioxidant butylated hydroxy toluene (BHT) as positive control. The experiments were performed in triplicates and scavenging activity was expressed as percentage inhibition, using the following formula:

(1) $$\begin{array}{c} \%\text{Inhibition}=\left[\frac{\left({\text{Abs}}_{\text{control}}-{\text{Abs}}_{\text{samples}}\right)}{{\text{Abs}}_{\text{control}}}\right]\times 100. \end{array}$$

### 2.6. *In Vitro* *α*-Amylase Inhibition Assay

The *α*-amylase inhibitory activity was determined as described in Jayasri et al. [28] with minor modifications. Briefly, 250 *μ*L of algal extracts with varying concentrations (250–1000 *μ*g/mL) and 250 *μ*L of 0.02 M sodium phosphate buffer (pH 6.9 with 0.006 M sodium chloride) containing *α*-amylase (porcine pancreatic *α*-amylase, Sigma, St. Louis, USA) solution (0.5 mg/mL) were incubated for 10 min at 25°C. After preincubation, 250 *μ*L of 1% starch solution in 0.02 M sodium phosphate buffer (pH 6.9 with 0.006 M sodium chloride) was added to each tube at 5 sec intervals. The reaction mixtures were then incubated at 25°C for 10 min. The reaction was stopped with 500 *μ*L dinitrosalicylic acid (Sigma, St. Louis, USA) colour reagent. The tubes were then incubated in a boiling water bath for 5 min and cooled to room temperature. The reaction mixture was then diluted by adding 5 mL of distilled water, and absorbance was measured at 540 nm in the UV-visible spectrophotometer (Systronics AU-2700, India). The experiments were performed in triplicate and the *α*-amylase inhibitory activity was calculated as percentage inhibition, using the formula

(2) $$\begin{array}{c} \%\text{Inhibition}=\left[\frac{\left({\text{Abs}}_{\text{control}}-{\text{Abs}}_{\text{samples}}\right)}{{\text{Abs}}_{\text{control}}}\right]\times 100. \end{array}$$

### 2.7. *In Vitro* *α*-Glucosidase Inhibition Assay

The *α*-glucosidase inhibitory activity was determined as described by Jayasri et al. [28] with slight modifications. Briefly, 50 *μ*L of algal extracts with varying concentrations (250–1000 *μ*g/mL) and 100 *μ*L of 0.1 M phosphate buffer (pH-6.9) containing *α*-glucosidase (SRL, India) solution (1.0 U/mL) were incubated in 96-well plates at 25°C for 10 minutes. After preincubation, 50 *μ*L of 5 mM p-nitrophenyl *α*-D-glucopyranoside (Sigma, St. Louis, USA) in 0.1 M phosphate buffer (pH-6.9) was added to each well at 5 sec intervals. The reaction mixture was then incubated at 25°C for 5 min. After incubation, absorbance readings were recorded at 405 nm using a plate reader (Bio-TEK, USA) and compared to the control which contained 50 *μ*L of buffer solution in place of algal extract. The experiments were performed in triplicate and the *α*-glucosidase inhibitory activity was calculated as percentage inhibition. The percentage of enzyme inhibition was calculated as follows:

(3) $$\begin{array}{c} \%\text{Inhibition}=\left[\frac{\left({\text{Abs}}_{\text{control}}-{\text{Abs}}_{\text{samples}}\right)}{{\text{Abs}}_{\text{control}}}\right]\times 100. \end{array}$$

### 2.8. *In Vitro* Dipeptidyl Peptidase-IV (DPP-IV) Inhibition Assay

DPP-IV inhibitory activity was determined according to the method of Al-Masri et al. [29]. Standard diprotin A (Sigma, St. Louis, USA) was diluted to various concentrations (0.2, 0.4, 0.8, 1.6, 3.2, and 6.4 *μ*g/mL) using Tris-HCl buffer (50 mM, pH 7.5) and the final volume was made to 35 *μ*L and DPP-IV enzyme (15 *μ*L) (Sigma, St. Louis, USA) was added to the above mixture. One unit of enzyme activity was defined as the amount of enzyme that catalyzes the release of 1 *μ*mol* para*-nitroaniline (pNA) from the substrate/min under assay conditions. After adding the enzyme, the mixture was preincubated for 10 min at 37°C to enhance the binding capacity of the inhibitor. This was followed by the addition of 50 *μ*L of (0.2 mM) Gly-pro-p-nitroanilide (Sigma, St. Louis, USA) in Tris-HCl as a substrate. Final incubation was done at 37°C for 30 min and the reaction was terminated by addition of 25 *μ*L of 25% glacial acetic acid. The absorbance was measured at 405 nm using microtiter plate reader (Bio-TEK, USA). Experiments were done in triplicate and the results obtained were compared with negative control.

### 2.9. DPP-IV Inhibition Assay of Seaweed Extract

Seaweed extracts were dissolved in dimethyl sulphoxide to make a stock concentration of 1000 *μ*g/mL. From the stock, the following concentrations were prepared (2.5, 10, 40, and 80 *μ*g/mL) in Tris-HCl buffer (50 mM, pH 7.5) in a total volume of 100 *μ*L/well. The assay was performed in triplicate according to standardized procedure of diprotin A. The percentage of DPP-IV inhibition was calculated as follows:

(4) $$\begin{array}{c} \%\text{Inhibition}=\left[\frac{\left({\text{Abs}}_{\text{control}}-{\text{Abs}}_{\text{samples}}\right)}{{\text{Abs}}_{\text{control}}}\right]\times 100. \end{array}$$

### 2.10. Maintenance of Cell Line

J774 cell line (mouse macrophage) was obtained from National Centre for Cell Science, Pune, India. The cells were grown in Dulbecco's Modified Eagle's Medium (DMEM) supplemented with 10% (v/v) heat-inactivated foetal bovine serum (FBS), 2 mM l-glutamine, and 100 U/mL of penicillin/streptomycin with 5% CO_2_ at 37°C in a humidified incubator. Exponentially growing cells were used for the experiment.

### 2.11. Cytotoxicity Assay

Briefly, cells were seeded at a density of 5 × 10^5^ cells/mL and allowed to attach for 24 h in 300 *μ*L of medium incubated at 37°C and 5% CO_2_. After 24 h, seaweed extracts were added to the medium at various concentrations (1000–250 *μ*g/mL) and incubated for 24 h. At the end of treatment, cells were incubated with MTT [3-(4, 5-dimethylthiazol-2-yl)-2, 5-diphenyltetrazolium bromide] (5 mg/mL) for 4 h. The formazan crystals formed were dissolved in DMSO (dimethyl sulfoxide) after aspirating the medium. The extent of cytotoxicity was measured spectrophotometrically at 630 nm with a microplate reader (Bio-TEK, USA).

### 2.12. DNA Fragmentation Assay

Acridine orange-ethidium bromide staining was used to characterize the morphological changes and apoptotic body formation, assessed by fluorescent microscopy. Briefly, J774 cells (5 × 10^5^) were seeded on sterile glass cover slips in six-well tissue culture plates and treated with test extracts (1000 *μ*g/mL) for 24 h. The medium was removed and washed twice with PBS (phosphate-buffered saline). Cells grown on cover slips were loaded with acridine orange-ethidium bromide (4 *μ*g/mL) of ratio (1 : 1) and incubated for 15 min at 37°C in the dark. Cells were viewed under Weswox LED fluorescent microscope (FM-3000) attached with a camera, and photographs were taken under 40 and 100x magnification at fluorescent conditions.

### 2.13. Haemolytic Assay

The seaweed extracts were evaluated for their haemolytic activity as described by Malagoli [30]. The blood sample was collected from a healthy volunteer (A^+ve^), using ethylene diamine tetra acetic acid (EDTA) as an anticoagulant. Whole blood was washed thrice in 9 volumes of sterile 0.9% NaCl saline solution; after each washing, the cells were centrifuged (150 ×g for 5 min) and the supernatant was discarded. The final pellet obtained was diluted, 1/9 (v/v) in sterile 0.9% NaCl saline solution and then 1/24 (v/v) in sterile Dulbecco's phosphate buffer saline (D-PBS), pH 7.0, containing 0.5 mM boric acid and 1 mM calcium chloride. The haemolytic activity of seaweed extracts was tested under* in vitro* conditions in 96-well plates. Briefly, each well received 100 *μ*L of 0.85% NaCl solution containing 10 mM CaCl_2_. 100 *μ*L of normal saline served as negative control, 0.1% Triton X-100 as a positive control, and test extracts of various concentrations (250–1000 *μ*g/mL) were added to each well. Then each well received 100 *μ*L of human erythrocytes diluted in D-PBS. After 30 min incubation at room temperature, the samples were centrifuged and supernatant was used to measure the absorbance of liberated haemoglobin at 630 nm [31].

### 2.14. GC/MS Analysis

GC/MS (Perkin Elmer, Clarus 680 GC coupled to a Clarus 600 MS) analysis was performed for the detection of major compounds present in various extracts of* T. ornata*. Column used in GC/MS was Elite-5MS (30.0 m, 0.25 mmID, and 250 *μ*mdf). Carrier gas used was helium at a constant flow rate of 1 mL/min. EI mode with electron energy set at 70 eV was used. 1 *μ*L of extract diluted with methanol was injected into GC/MS and the compounds were identified based on the molecular structure, molecular mass, and calculated fragment ratio of resolved spectra with that of mass spectra available from the library. Spectral data were interpreted using the database of National Institute Standard And Technology (NIST).

### 2.15. Statistical Analysis

All the data reported were expressed as mean ± S.E.M. Statistical analysis was performed using two-way ANOVA. The values were considered to be significantly different when the *P* value was <0.0001 compared to the baseline values. Software employed for statistical analysis was Graph-Pad Prism, Version 5.

## 3. Results

### 3.1. Identification of Collected Seaweeds

**Figure 1:**
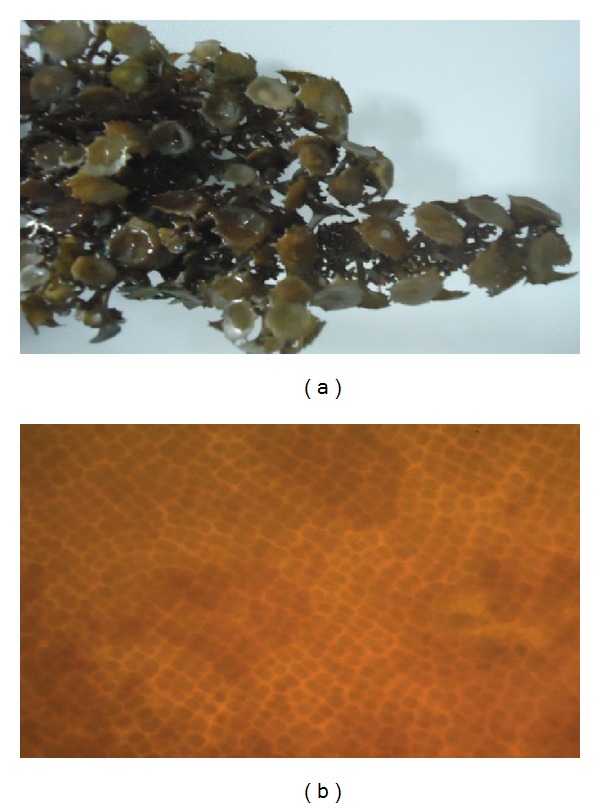
(a) Macroscopic view of* Turbinaria ornata* collected from Mandapam coastal region in Gulf of Mannar, Tamilnadu, India. Stiff erect stalks, hard thick leaves (blades) characterized by lateral ridges, and outer marginal blade with stiff row of spines are the unique characteristics of this species. (b) Microscopic image of* Turbinaria ornata* (magnification 40x).

The samples were identified based on the morphological characteristics in response to environmental conditions: stiff erect stalks, hard thick leaves (blades) characterized by lateral ridges, and outer marginal blade with stiff row of spines (Figure 1). Based on the above characters, seaweed species was identified as* T. ornata* and authenticated by Dr. P. Kaladharan principle scientist and scientist in charge, Calicut Regional Centre of Central Marine Fisheries Research Institute.

### 3.2. Phytochemical Analysis of* T. ornata*

Qualitative analysis of the crude extracts of* T. ornata* showed the presence of major phytochemicals like alkaloids, phenols, flavanoids, proteins, lipids, carbohydrates, glycosides, tannins, and saponins in all the studied extracts (Table 1).

### 3.3. 2,2-Diphenyl-1-picrylhydrazyl (DPPH) Radical Scavenging Activity

**Figure 2:**
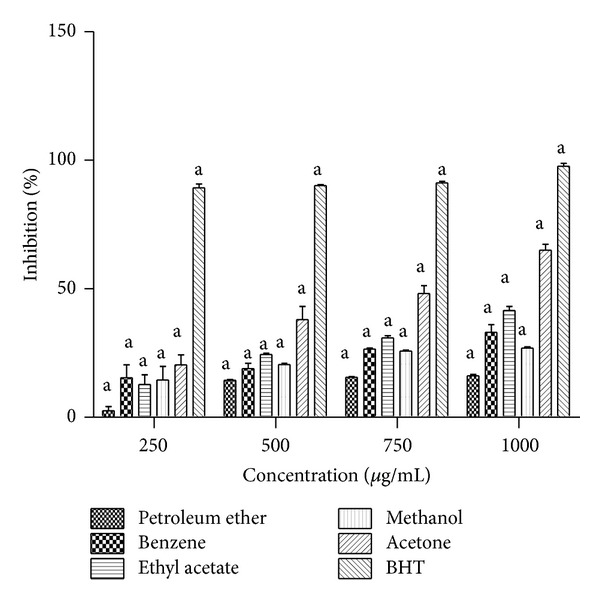
DPPH radical scavenging activity of petroleum ether, benzene, ethyl acetate, methanol, and benzene extracts of* Turbinaria ornata*. BHT is used as positive control and absorbance was measured at 517 nm. Values are means ± SD (*n* = 3) (^a^ *P* < 0.0001 considered as significant).

The antioxidant potential of* T. ornata* was studied through its scavenging ability of the stable radical DPPH. Acetone extract showed significant scavenging ability on DPPH (65%) at a concentration of 1000 *μ*g/mL when compared with that of a standard BHT (97%). However, none of the extracts exhibited higher activity than BHT at the same concentration (Figure 2).

### 3.4. *In Vitro* *α*-Amylase Inhibition Assay

**Figure 3:**
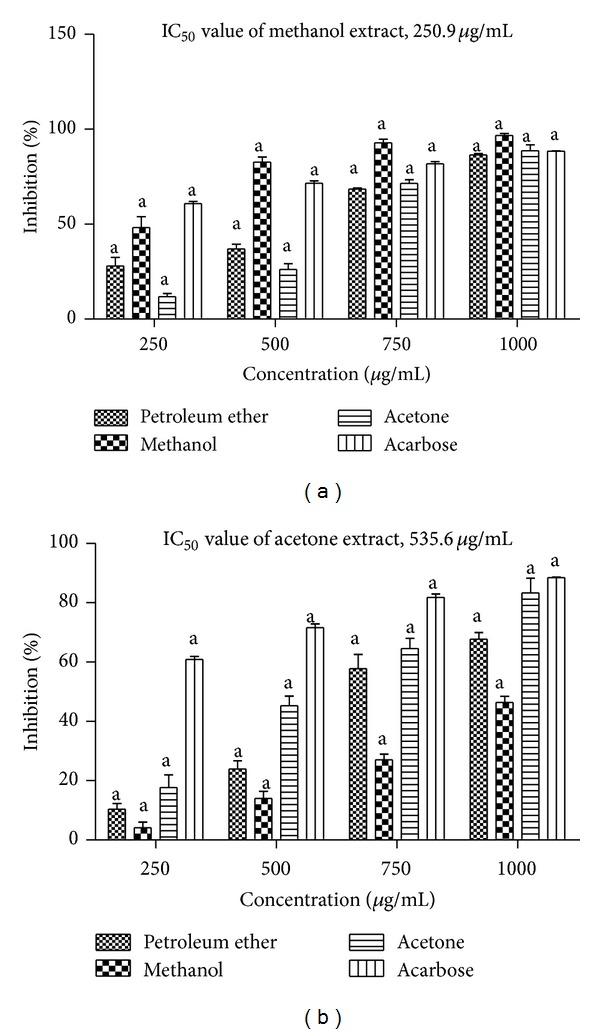
(a)* In vitro* *α*-amylase inhibitory activity of petroleum ether, methanol, and acetone extracts of* Turbinaria ornata*. Absorbance was measured at 540 nm. (b)* In vitro* *α*-glucosidase inhibitory activity of petroleum ether, methanol, and acetone extracts of* Turbinaria ornata*, acarbose is used as positive control and absorbance was measured at 405 nm. Values are means ± SD (*n* = 3) (^a^ *P* < 0.0001 considered significant).

Inhibitory effect of* T. ornata* on *α*-amylase is shown in Figure 3(a). Among the five extracts studied (petroleum ether, benzene, ethyl acetate, methanol, and acetone), three extracts showed (petroleum ether, methanol, and acetone) significant *α*-amylase inhibition at all the tested concentrations (250, 500, 750, and 1000 *μ*g/mL). Methanol extract of* T. ornata* showed maximum inhibition of 96.5% with an IC_50_ of 250.9 *μ*g/mL followed by acetone extract (87.6%) at a concentration of 1000 *μ*g/mL.

### 3.5. *In Vitro* *α*-Glucosidase Inhibition Assay

The *α*-glucosidase inhibitory activity of* T. Ornata* extracts was determined using p-nitrophenyl *α*-D-glucopyranoside as a substrate and these were compared with a standard acarbose (88%) (Figure 3(b)). At a concentration of 1000 *μ*g/mL, acetone extract showed maximum inhibition (87.6%) with an IC_50_ value of 535.6 *μ*g/mL. The inhibitory activity of acetone extracts was found to be more potent and similar when compared to the positive control acarbose.

### 3.6. *In Vitro* Dipeptidyl Peptidase-IV (DPP-IV) Inhibition Assay

**Figure 4:**
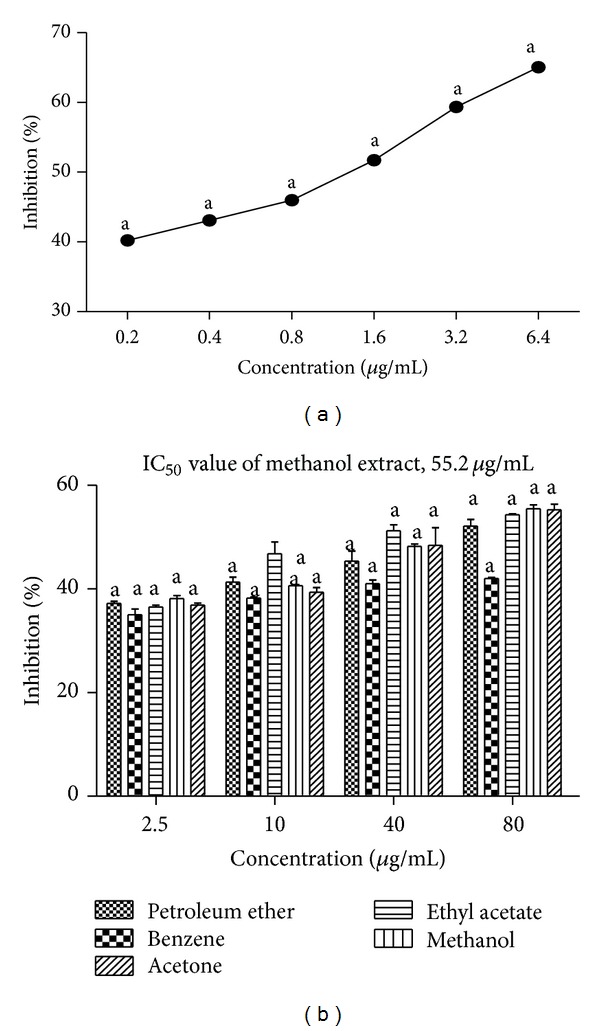
(a) DPP-IV inhibitory activity of diprotin A as positive control. (b) DPP-IV inhibitory activity of petroleum ether, benzene, ethyl acetate, methanol, and acetone extracts of* Turbinaria ornata*. Absorbance was measured at 405 nm. Values are means ± SD (*n* = 3) (^a^ *P* < 0.0001 considered significant).

*T. ornata* extracts were evaluated for their mode of action to stimulate insulin secretion through inhibition of DPP-IV enzyme. The effectiveness of various extracts was evaluated on the basis of percentage inhibition and IC_50_ values obtained. Among the five different solvent extracts (petroleum ether, benzene, ethyl acetate, methanol, and acetone), methanol extracts showed maximum percentage inhibition of 55.4% with an IC_50_ value of 55.2 *μ*g/mL at a maximum concentration of 80 *μ*g/mL. All the results were compared with a standard diprotin A (Figure 4).

### 3.7. Cytotoxic Effect of* T. ornata* Extracts on J774 Cell Line

**Figure 5:**
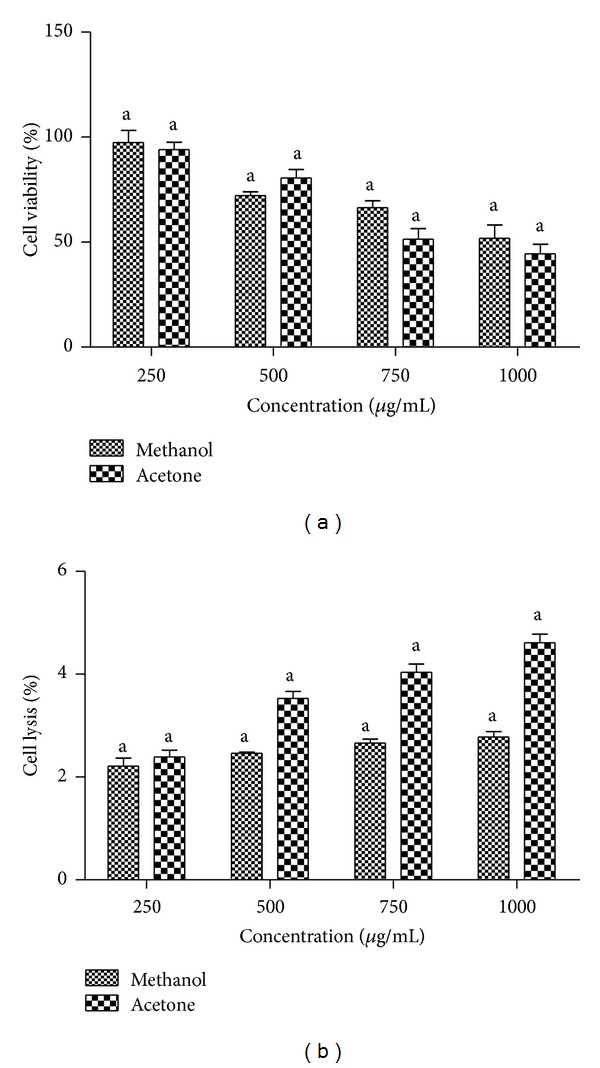
(a) Cell viability determined by MTT assay, J774 cells treated with methanol and acetone extracts of* Turbinaria ornata*. Absorbance was measured at 630 nm. (b) Haemolytic activity of methanol and acetone extracts of* Turbinaria ornata*. Absorbance was measured at 630 nm. Values are means ± SD (*n* = 3) (^a^ *P* < 0.0001 considered significant).

To evaluate the cytotoxic effect of the extracts, J774 cell line was incubated for 24 h with various concentrations of extracts. As shown in Figure 5(a), the decrease in viability correlates with the increase in concentration. MTT assay indicates that J774 cells treated with various extracts of* T. ornata* were safe and did not show any toxic effect against the cell treated at a lower concentration of 250 *μ*g/mL. The observed cytotoxicity was found to be 2.57% when treated with methanol extract of* T. ornata*. 50% of cells were found to be viable when treated with a higher concentration of 1000 *μ*g/mL.

### 3.8. DNA Fragmentation Assay

**Figure 6:**
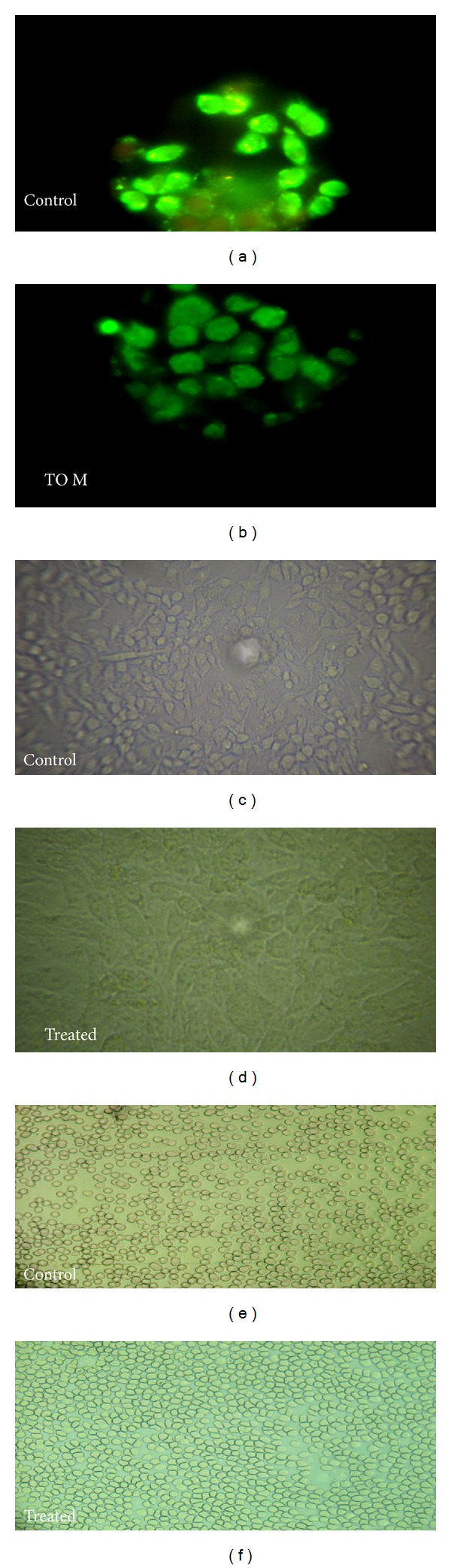
(a) Fluorescent photomicrographs (100x magnification) of J774 cells treated without seaweed extracts. (b) J774 cells treated with* Turbinaria ornata* methanol extract (TO M). (c) Phase contrast microscopic image of J774 cells treated with media alone (control). (d) Phase contrast microscopic image of J774 cells treated with test extracts. ((e)-(f)) Microscopic image (40x magnification) of* in vitro* haemolytic activity.

Staining cells with acridine orange-ethidium bromide is used to measure the proportion of viable, apoptotic, and necrotic cells. J774 cells treated with a maximum concentration of 1000 *μ*g/mL of algal extracts were found to be viable and there was no change in cellular morphology including chromatin condensation, membrane blebbing, and fragmented nuclei (Figure 6).

### 3.9. Haemolytic Assay

The lead extracts methanol (2.78%) and acetone (4.61%) were found to be nontoxic at a dosage of 1000 *μ*g/mL (Figure 5(b)). Also, the extracts did not show any erythrocyte membrane damage at all the tested concentrations.

### 3.10. GC-MS Analysis of the Acetone Extract of* T. ornata*

**Figure 7:**
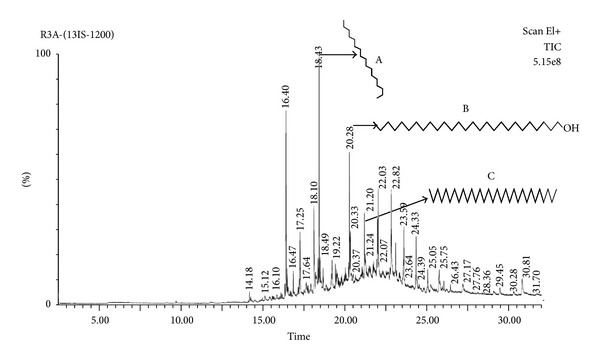
Gas chromatogram of acetone extract of* Turbinaria ornata* (A) 8-heptadecene, (B) 1-heptacosanol, and (C) hentriacontane.

The crude extracts were analyzed by GC-MS and compared with the retention time (RT) and mass spectra available in the data library of NIST. The GC-MS analysis reveals the presence of major bioactive compounds like hentriacontane, z, z-6, 28-heptatriactontadien-2-one, 8-heptadecene, and 1-heptacosanol. Chromatograms obtained were shown in Figure 7.

## 4. Discussion

Marine seaweeds are good source of dietary fibres, polysaccharides, polyphenols, polyunsaturated fatty acids, minerals, and vitamins which possess various biological activities like antidiabetic, anti-inflammatory, and antioxidant activity [32]. Although this evidence seems to be valid, detailed research should be carried out to ensure the presence of various phytochemicals present in the seaweed extracts. In the current study, marine seaweed* T. ornata* was extracted and screened for both nonpolar and polar compounds using various solvents based on polarity. Qualitative analysis showed the presence of major bioactive compounds like alkaloids, phenols, flavanoids, glycosides, lipids, carbohydrates, proteins, tannins, oils, and fats. Presence of these bioactive molecules in various extracts of this seaweed may be responsible for various biological activities (antiviral, anti-inflammatory, and anticoagulant) [17–19].

The objective of this study was to investigate the antidiabetic and antioxidant potential of marine seaweed* T. ornata* and to enumerate major phytocompounds present in the extracts. One of the major causes for diabetes is due to the increased production of reactive oxygen species or impaired antioxidant defense system which leads to oxidative damage of beta cells [33]. In this study, acetone extract of* T. ornata* showed significant scavenging capability against DPPH (65%) at a maximum concentration of 1000 *μ*g/mL compared with a positive control BHT (97%). As previously reported by Ananthi et al. and Chattopadhyay et al. [23, 25], the fucoidan fraction of* Turbinaria conoides* (61%) at a concentration of 1000 *μ*g/mL and aqueous extract of* T. ornata* (80%) at a concentration of 500 *μ*g/mL showed the similar scavenging capability on DPPH radicals. Our results were similar to these reports which confirm the antioxidant activity of* T. ornata*.

In the present study, the edible seaweed* T. ornata* was evaluated for* in vitro* *α*-amylase, *α*-glucosidase, and DPP-IV inhibitory property. Among the five extracts studied, petroleum ether, acetone, and methanol extracts showed significant percentage inhibition. Methanol extract of* T. ornata* showed maximum percentage inhibition on *α*-amylase (96.5%) with an IC_50_ value of 250.9 *μ*g/mL with more inhibition than positive control acarbose (88%). Acetone extract of* T. ornata* showed more potent inhibition on *α*-glucosidase (84.3%) with an IC_50_ value of 535.6 *μ*g/mL at a concentration of 1000 *μ*g/mL, similar to the positive control acarbose (88%). The DPP-IV inhibitory potential of methanol extract of* T. ornata* showed (55.4%) inhibition with an IC_50_ value of 55.2 *μ*g/mL which is comparable with that of positive control diprotin A (65%). Similar findings were shown by two brown seaweeds* Ecklonia stolonifera* Okumura and* Eisenia bicyclis* [34]. Several studies reported that the *α*-amylase and *α*-glucosidase inhibitory action of the class Phaeophyceae [35, 36] supports that these algal species are excellent source for antidiabetic agents. Apart from *α*-amylase and *α*-glucosidase,* T. ornata* extracts also showed significant DPP-IV inhibition. Based on these results, this seaweed can be used as alternative therapy for diabetes by raising the levels of endogenous incretin hormones (GLP-1 and GIP).

In this study, we also investigated the effects of crude extracts (methanol, acetone) of* T. ornata* in macrophage cell line (J774). The cytotoxic effects of the extracts were determined by using an MTT assay. After 24 h exposure, 250–1000 *μ*g/mL of methanol and acetone extracts significantly decreased cell viability by increasing concentration. As previously reported, seaweed extracts which have higher phenol content may explain the level of cytotoxicity at higher concentrations (1000 *μ*g/mL) [36, 37], whereby up to 500 *μ*g/mL of seaweed extracts did not show any effect on J774 cell viability, and more than 90% of cells were found to be viable at a concentration of 250 *μ*g/mL. Staining cells with fluorescent dyes is used to differentiate the nuclear morphology of live and apoptotic cells. J774 cells were analyzed in the presence of acridine orange and ethidium bromide to determine the nuclear morphology of viable and necrotic cells. Cells stained green represent viable cells and ethidium bromide selectively stains the cells that have lost membrane integrity and stains DNA orange. In this study acridine orange-ethidium bromide staining revealed that there was no change in cellular morphology, including chromatin condensation, membrane blebbing, and fragmented nuclei occurring due to the treatment of extracts even at higher concentration (1000 *μ*g/mL). Our results indicate that the tested extracts did not induce haemolysis at a concentration of (1000 *μ*g/mL). Based on the above results the methanol and acetone extracts of* T. ornata* were found to be nontoxic under* in vitro* conditions.

GC-MS analysis revealed the presence of major bioactive compounds like hentriacontane, z, z-6, 28-heptatriactontadien-2-one, 8-heptadecene, and 1-heptacosanol. Hentriacontane is the major component present in* Oldenlandia diffusa*, a natural source for the treatment of cancer in Asia [38], and also has reported activities like anti-inflammatory and antioxidant properties [38, 39]. Currently, there is no information regarding the antidiabetic effects of hentriacontane. Further, purification and characterization of active compounds along with mechanism studies will only give conclusive data on the antidiabetic property of tested algae.

## 5. Conclusions

The present study indicates that crude extracts of* T. ornata* possess* in vitro* *α*-amylase, *α*-glucosidase, and DPP-IV inhibitory activity. Due to their strong enzyme inhibitory activity, they are expected to suppress the glycemic response in diabetic conditions. Additionally, the extracts are capable of scavenging free radicals;* in vitro* toxicological parameters indicate that the IC_50_ of methanol and acetone extracts of* T. ornata* for *α*-amylase, *α*-glucosidase, and DPP-IV inhibition are greatly below cytotoxic levels. Future studies on* in vivo* antidiabetic activity of selected extracts and identification of major compounds responsible for antidiabetic action are in progress. These studies will reveal the possible antidiabetic mechanisms of* T. ornata*.

## Figures and Tables

**Table 1:** Qualitative phytochemical screening of various extracts of *Turbinaria ornata *(+: present, −: absent).

Phytochemicals	Pet ether	Benzene	Ethyl acetate	Methanol	Acetone
Alkaloids	+	+	+	+	+
Phenols	+	+	+	+	+
Flavanoids	+	+	+	+	+
Proteins and amino acid	+	+	+	+	+
Lipids	+	+	+	+	+
Carbohydrates	+	+	+	+	+
Saponins	−	−	+	−	−
Glycosides	−	−	+	+	−
Tannins	+	+	+	−	−
Oils and fats	+	−	−	−	−
